# Potential Prognostic and Metastatic Implications of MACC1 and MMP8 in Colorectal Cancer

**DOI:** 10.3390/cimb48050496

**Published:** 2026-05-11

**Authors:** Hilal Oğuz Soydinç, Sena Şen, Murat Serilmez, Senem Karabulut

**Affiliations:** 1 Department of Basic Oncology, Oncology Institute, Istanbul University, 34093 Istanbul, Türkiye; sena.sen@istanbul.edu.tr (S.Ş.); murat.serilmez@istanbul.edu.tr (M.S.); 2 Department of Clinic Oncology, Oncology Institute, Istanbul University, 34093 Istanbul, Türkiye; senem.karabulut@istanbul.edu.tr

**Keywords:** biomarker, colorectal cancer, MACC1, MMP8, prognosis

## Abstract

Colorectal cancer (CRC) remains a major cause of cancer-related morbidity and mortality worldwide. Metastasis regulators and matrix metalloproteinases have been implicated in tumor progression; however, their clinical significance in CRC remains incompletely defined. In this study, the prognostic value of MACC1 and MMP8 expression levels was investigated. A total of 140 patients diagnosed with CRC and 48 healthy controls were included. Serum levels of MACC1 and MMP8 were measured using ELISA. Clinicopathological parameters were recorded, and their associations with biomarker expression were analyzed. Both MACC1 and MMP8 levels demonstrated moderate diagnostic performance with comparable area under the curve values. A strong positive correlation between MACC1 and MMP8 expression was observed. MACC1 expression was significantly associated with metastasis status and tumor stage, whereas MMP8 expression was associated with tumor localization. In survival analyses, established clinicopathological factors, particularly tumor stage and metastasis status, were identified as the primary determinants of overall survival. In multivariate analysis, tumor stage remained the only consistent independent prognostic factor, while MMP8 showed a modest independent association in a separate model. MACC1 did not retain independent prognostic significance. Although MACC1 and MMP8 may have diagnostic and biological relevance in CRC, their prognostic utility appears limited compared to established clinical parameters. Further large-scale prospective studies are needed.

## 1. Introduction

Colorectal cancer (CRC) is the third most frequently diagnosed malignancy worldwide, affecting over 1.2 million individuals each year [1]. The overall five-year survival rate is approximately 64.9%, but it drops significantly to about 13.1% in patients with metastatic disease [2]. Although colonoscopy is the gold standard for early detection, its invasive nature limits its use in large-scale screening. Commonly used biomarkers such as CEA and CA19-9 lack sufficient sensitivity and specificity for reliable early diagnosis and prognosis. Molecular alterations, including RAS and BRAF mutations, are associated with poor prognosis and are primarily used in the clinical management of metastatic cases [3].

In this context, identifying novel and more reliable molecular biomarkers has become increasingly important. Metastasis-Associated in Colon Cancer 1 (MACC1) was initially identified as a direct regulator of the hepatocyte growth factor receptor and is recognized as a key transcription factor involved in tumor development and metastasis in CRC [4]. MACC1 contributes to multiple oncogenic processes, including proliferation, migration, invasion, apoptosis, and metastasis, and is considered a prognostic biomarker in various cancers, including CRC [5]. Furthermore, MACC1 expression can be detected not only in tumor tissues but also in peripheral blood samples, serving as a minimally invasive liquid biopsy marker [6]. The presence of circulating MACC1 transcripts is significantly associated with poor survival outcomes, consistent with findings from tumor tissue analyses in glioblastoma [7], lung [8], breast [9,10], ovarian [11,12], and colorectal cancers [13,14,15]. These findings highlight the potential of MACC1 as a non-invasive prognostic biomarker and support its further investigation in clinical settings.

Despite advances in surgical techniques and perioperative care for CRC, anastomotic leakage remains a major postoperative complication [16]. Early detection of this condition is challenging, underscoring the need for reliable predictive biomarkers. Anastomotic integrity is closely linked to extracellular matrix (ECM) remodeling, particularly collagen degradation by matrix metalloproteinases (MMPs) [17]. In the early postoperative period, increased MMP activity and delayed ECM synthesis may lead to reduced anastomotic strength [16]. Experimental and clinical evidence shows that elevated levels of MMP-1, MMP8, and MMP-9 are associated with a higher risk of anastomotic leakage, whereas inhibition of MMP activity may enhance tissue integrity [18]. Therefore, MMPs are considered both potential early biomarkers and therapeutic targets for anastomotic healing.

Given the limitations of current biomarkers in prognostic evaluation of CRC, there is an increasing need for more reliable molecular indicators. In this study, serum levels of MMP8 and MACC1 were measured in patients with CRC in a Turkish population. The aim was to assess the potential prognostic value of these molecules for disease detection and clinical outcome prediction.

## 2. Materials and Methods

### 2.1. Patients and Sample Collection

This study was designed based on a priori power analysis using the G*Power 3.1.9.7 program (Heinrich-Heine-Universität Düsseldorf, Germany) [19]. The power analysis (α = 0.05, 1 − β = 0.80, two-tailed, Cohen’s d = 0.5) indicated that a total of 144 participants were required, including 96 patients and 48 controls (df = 142), to detect a statistically significant difference between the groups. For the study population, archived serum samples were retrospectively collected from 140 patients diagnosed with CRC. Additionally, a control group of 48 healthy individuals with comparable age and gender distribution and no history of malignancy was included. The study protocol was approved by the institutional ethics committee, and all procedures followed the Declaration of Helsinki. Written informed consent was obtained from all participants before inclusion. Tumor staging was determined according to the criteria established by the American Joint Committee on Cancer and the Union for International Cancer Control. Surgical eligibility was assessed based on tumor stage, patient performance status, and multidisciplinary clinical evaluation in accordance with current oncological guidelines.

The mean age of the patients was 60.13 ± 11.62 years (median: 60.00; range: 24–84). The study cohort included 140 patients, with 84.3% (*n* = 118) older than 50 years and 15.7% (*n* = 22) younger than 50 years. Male patients predominated (68.6%, *n* = 96), while females accounted for 31.4% (*n* = 44). Smoking was reported in 43.6% (*n* = 61) and alcohol consumption in 18.6% (*n* = 26) of patients. Comorbid conditions were present in 40.0% (*n* = 56), and a positive family history of cancer was identified in 30.7% (*n* = 43), with 10.0% (*n* = 14) specifically reporting a family history of CRC. Performance status, assessed by ECOG, was predominantly favorable: 50.7% (*n* = 71) were classified as ECOG 0 and 43.6% (*n* = 61) as ECOG 1, while only a small proportion had poorer performance (ECOG ≥ 2). Tumor localization was most frequently in the rectum (37.9%, *n* = 53) and sigmoid colon (26.4%, *n* = 37); overall, tumors were more commonly located in the colon (57.9%, *n* = 81) than in the rectum (42.1%, *n* = 59). For surgical management, low anterior resection (LAR) was the most common procedure (42.9%, *n* = 60), followed by colectomy (40.0%, *n* = 56). Intestinal obstruction was present in 12.1% (*n* = 17) of patients. Histopathological evaluation showed adenocarcinoma as the predominant subtype (92.1%, *n* = 129), with mucinous histology in 7.9% (*n* = 11). Lymph node metastasis was identified in 28.6% (*n* = 40) of patients. The mean percentage of positive lymph nodes was 6.39 ± 15.43 (median: 0.00; range: 0.0–85.7). Distant metastasis was detected in 42.1% (*n* = 59), most commonly involving the liver (28.6%, *n* = 40), followed by other sites (13.6%, *n* = 19). Among metastatic cases, synchronous metastases were observed in 24.3% (*n* = 34) and metachronous metastases in 17.9% (*n* = 25). Regarding disease stage, most patients were diagnosed at advanced stages: 45.7% (*n* = 64) at stage III and 42.1% (*n* = 59) at stage IV, while 12.1% (*n* = 17) were at stage II. At the last follow-up, 77.9% (*n* = 109) of patients were alive, while 22.1% (*n* = 31) had died. The mean overall survival time was 14.13 ± 7.62 months (median: 14.00; range: 1–34).

Peripheral venous blood samples (~5 mL) were collected from each participant prior to the initiation of any treatment into serum separator tubes with a clot activator. Samples were centrifuged at 3000× *g* for 10 min to separate the serum fraction. The serum was carefully aliquoted into sterile microtubes to avoid repeated freeze–thaw cycles and stored at −80 °C until further analysis.

### 2.2. Determination of MACC1 and MMP8 Levels by Enzyme-Linked Immunosorbent Assay (ELISA)

MACC1 (Catalog No: E3853Hu) and MMP8 (Catalog No: E0903Hu) concentrations were quantified using a commercially available colorimetric ELISA kit (Bioassay Technology Laboratory, Shanghai, China) in accordance with the manufacturer’s instructions. Peripheral blood samples were allowed to clot at room temperature for approximately 10–20 min and were subsequently centrifuged (2000–3000 rpm, 20 min) to obtain serum. The supernatant was carefully collected to avoid cellular contamination and aliquoted into sterile tubes. Samples were stored at −80 °C until analysis, and repeated freeze–thaw cycles were avoided; all measurements were performed using samples that had undergone a single thaw. Prior to the assay, samples and reagents were equilibrated to room temperature and gently mixed. ELISA measurements were conducted in accordance with a standardized workflow. A serial dilution (1:2) of the provided standard solution was used to generate a multi-point calibration curve. The optical density (OD) values corresponding to each standard concentration were used to construct the standard curve, which was fitted using the most appropriate regression model. Sample concentrations were calculated by interpolation from this curve. Assays were carried out using pre-coated 96-well plates. Following incubation with serum samples and detection reagents, wells were washed five times using a wash buffer prepared from a 25× stock solution. Plate washing was performed using a manual washing procedure. After substrate addition and incubation under controlled conditions, the enzymatic reaction was stopped, and absorbance was measured at 450 nm within 10 min using a microplate spectrophotometer (Multiskan™ GO, Thermo Scientific, Waltham, MA, USA). All samples were analyzed in duplicate within the same batch to minimize inter-assay variability. Cases and controls were processed in mixed and blinded batches to reduce potential measurement bias. The intra-assay and inter-assay coefficients of variation were below 8% and 10%, respectively, indicating acceptable assay precision. Samples with missing or technically inadequate measurements were excluded from the final analysis. The assay sensitivity (limit of detection, LOD) was <0.1 ng/mL for MMP-8 and 0.021 ng/mL for MACC1, as specified by the manufacturer. The analytical sensitivity and dynamic range of the assays were consistent with the specifications provided by the manufacturer.

### 2.3. Statistical Analysis

All statistical analyses were performed using IBM SPSS Statistics software (version 31; IBM Corp., Armonk, NY, USA). Continuous variables were expressed as mean ± standard deviation (SD), median, and range, while categorical variables were summarized as frequencies and percentages. The normality of continuous variables was assessed using the Kolmogorov–Smirnov test. Comparisons between categorical variables were performed using the Pearson chi-square test or Fisher’s exact test, as appropriate. Correlations between continuous variables were evaluated using Pearson correlation analysis. Receiver operating characteristic (ROC) curve analysis was conducted to assess the diagnostic performance of MACC1 and MMP8 expression levels. The area under the curve (AUC), sensitivity, specificity, and optimal cut-off values were calculated. Overall survival (OS) was defined as the time from diagnosis to death or last follow-up. Survival curves were estimated using the Kaplan–Meier method and compared using the log-rank test. Mean survival times with standard errors and 95% confidence intervals (CIs) were reported. Univariate and multivariate survival analyses were performed using the Cox proportional hazards regression model to identify independent prognostic factors. Hazard ratios (HRs) with 95% CIs were calculated. Variables with clinical relevance or *p* < 0.10 in univariate analysis were included in the multivariate model. Separate multivariate Cox models were constructed to evaluate the independent prognostic effects of MACC1 and MMP8 by including each marker individually alongside clinicopathological variables. A two-sided *p*-value of <0.05 was considered statistically significant.

A stepwise modeling strategy was adopted for the multivariate Cox regression analysis in this study. In the initial stage, a comprehensive model including all clinicopathological and molecular variables was constructed. However, due to the limitation related to the number of events per variable and the potential risk of collinearity among the biomarkers (MACC1 and MMP8) or between these markers and tumor stage, separate models were subsequently developed. These models were designed to evaluate the independent prognostic value of each biomarker after adjustment for tumor stage and other key clinical variables. This strategy was pre-specified prior to analysis in order to maintain model stability and to minimize the risk of overfitting.

## 3. Results

**Figure 1:**
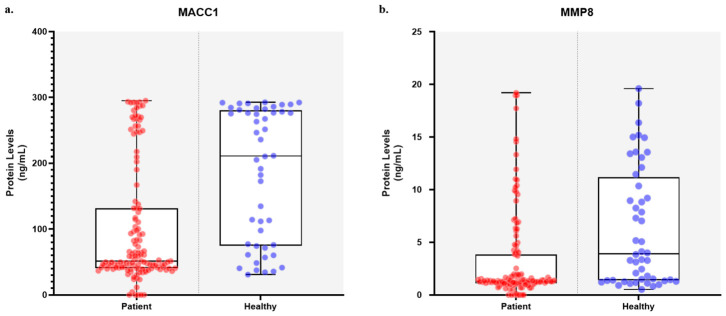
The distribution of (**a**) MACC1 and (**b**) MMP8 serum levels. Patients were stratified into low and high expression groups based on median values. Boxplots represent the median and interquartile range, while individual data points are overlaid to illustrate the distribution of samples.

The study cohort was evaluated for MACC1 and MMP8 expression. Figure 1 illustrates the distribution of MACC1 and MMP8 expression levels. The mean MACC1 expression level was 103.44 ± 91.13, with a median of 53.31 (range: 0.00–295.01). The mean MMP8 expression level was 3.05 ± 3.96, with a median of 1.27 (range: 0.00–19.21). Patients were dichotomized into low and high expression groups based on mean values. MACC1 expression was distributed with 70.0% (*n* = 98) classified as low and 30.0% (*n* = 42) as high. MMP8 expression was similarly distributed, with 72.9% (*n* = 102) in the low group and 27.1% (*n* = 38) in the high group. Correlation analysis was performed to assess the relationship between MACC1 and MMP8 expression levels. A strong, statistically significant positive correlation was found between MACC1 and MMP8 (r = 0.887, *p* < 0.001; Pearson correlation), indicating that increased MACC1 expression levels were associated with increased MMP8 expression levels in the study cohort.

The associations between MACC1 and MMP8 expression levels and clinicopathological parameters are summarized in Table 1. Statistically significant associations were found for MACC1 expression with metastasis status (*p* = 0.026), localization of metastasis (*p* = 0.039), and tumor stage (*p* = 0.044). High MACC1 expression was observed more frequently in non-metastatic patients, while low expression was more common in patients with metastatic disease. Differences in metastatic site distribution were also noted between MACC1 expression groups. For tumor stage, higher MACC1 expression was more often associated with stage II disease, whereas lower expression was more prevalent in advanced stages. For MMP8 expression, a statistically significant association was found only with tumor localization category (*p* = 0.007), with high MMP8 expression detected more frequently in colon tumors than in rectal tumors. No other clinicopathological parameters showed a significant relationship with MMP8 expression. Overall, most clinical variables were not significantly associated with either MACC1 or MMP8 expression (*p* > 0.05).

**Figure 2:**
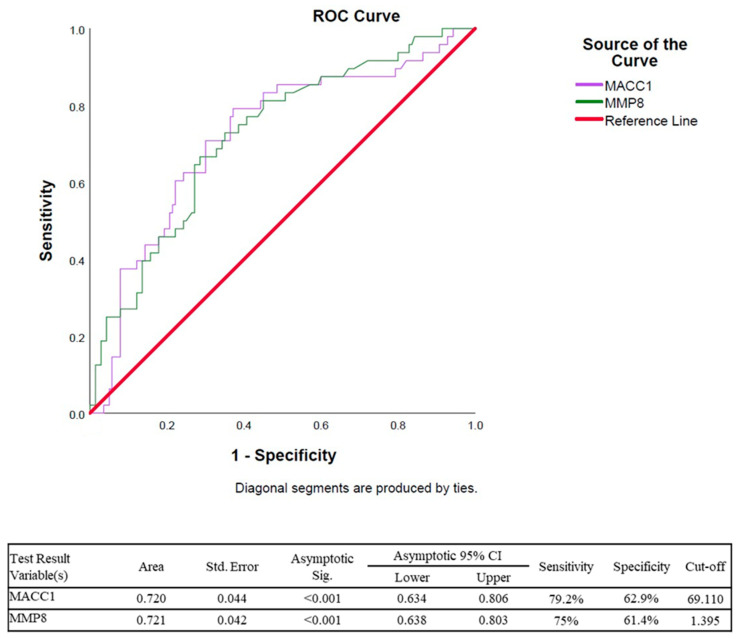
ROC curve analysis of MACC1 and MMP8 expression levels for discriminating between study groups. The diagonal red line represents the line of no discrimination, indicating random classification. Both MACC1 (purple curve) and MMP8 (green curve) exhibited curves above the reference line, indicating discriminatory capacity. The relatively overlapping ROC curves and confidence intervals suggest comparable diagnostic performance between the two biomarkers.

ROC curve analysis was conducted to assess the diagnostic performance of MACC1 and MMP8 expression levels, as shown in Figure 2. Both biomarkers showed statistically significant discriminatory ability (*p* < 0.001). The AUC for MACC1 was 0.720 (95% CI: 0.634–0.806), indicating moderate diagnostic performance, with a sensitivity of 79.2% and specificity of 62.9% at a cut-off value of 69.110. MMP8 had an AUC of 0.721 (95% CI: 0.638–0.803), with a sensitivity of 75.0% and specificity of 61.4% at a cut-off value of 1.395. Both MACC1 and MMP8 demonstrated comparable and moderate diagnostic accuracy, with overlapping confidence intervals, suggesting similar predictive capacities for distinguishing the study groups.

**Figure 3:**
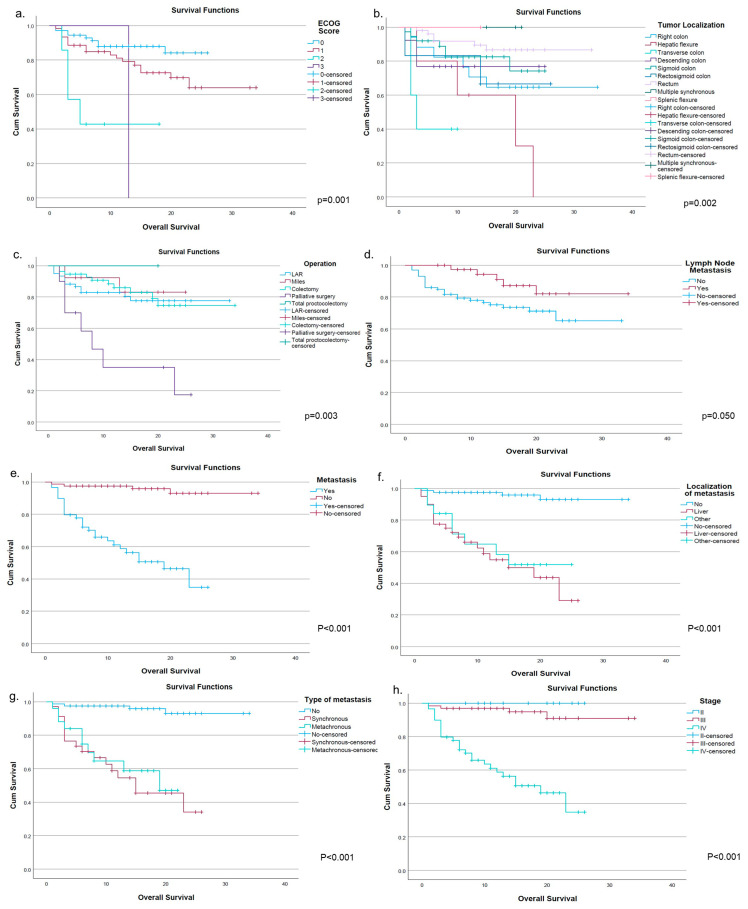
Kaplan–Meier curves according to clinicopathological parameters. Kaplan–Meier survival analyses demonstrating the impact of significant clinical variables on overall survival are presented. (**a**) ECOG performance status, (**b**) tumor localization, (**c**) type of operation, (**d**) lymph node metastasis, (**e**) presence of distant metastasis, (**f**) localization of metastasis, (**g**) type of metastasis (synchronous vs. metachronous), and (**h**) TNM stage. Statistically significant differences between groups were assessed using the log-rank test, and corresponding *p*-values are indicated in each panel.

OS analyses revealed several clinically and statistically significant associations, as shown in both the Kaplan–Meier survival curves (Figure 3a–h) and the survival analysis Table 2. Among clinical parameters, ECOG performance status had a strong impact on survival (*p* = 0.001), with markedly reduced mean OS in patients with poorer performance scores (ECOG 2: 9.57 months) compared to ECOG 0–1 group. Tumor localization was another significant determinant (*p* = 0.029), with rectal tumors showing longer mean OS (29.15 months) compared to colon tumors (24.68 months), consistent with the separation observed in the Kaplan–Meier curves. Similarly, type of operation was significantly associated with OS (*p* = 0.003), as illustrated in the corresponding survival plot, suggesting that surgical approach may influence patient outcomes. Disease burden-related parameters demonstrated the strongest associations with survival. The presence of distant metastasis was significantly associated with poorer OS (15.86 vs. 32.48 months, *p* < 0.001), which is prominently visualized in the survival curves with clear stratification between metastatic and non-metastatic groups. Furthermore, metastasis localization (*p* < 0.001) showed that liver and other metastatic sites were associated with similarly reduced survival compared to non-metastatic patients. Likewise, type of metastasis (synchronous vs. metachronous) was significantly associated with OS (*p* < 0.001), with both groups demonstrating substantially worse survival than non-metastatic cases. Advanced disease stage also played a critical role. TNM stage was significantly associated with OS (*p* < 0.001), supported by the Kaplan–Meier curves showing progressively worse survival with increasing stage. Additionally, lymph node metastasis showed borderline statistical significance (*p* = 0.05), with patients with nodal involvement demonstrating different survival patterns, as suggested by the corresponding survival curve. In contrast, several demographic and clinicopathological variables, including age, gender, smoking status, alcohol consumption, comorbidity, family history, histopathology, and molecular markers (MACC1 and MMP8 status), were not significantly associated with OS (all *p* > 0.05). These variables showed overlapping survival distributions without meaningful separation in the Kaplan–Meier analyses.

**Figure 4:**
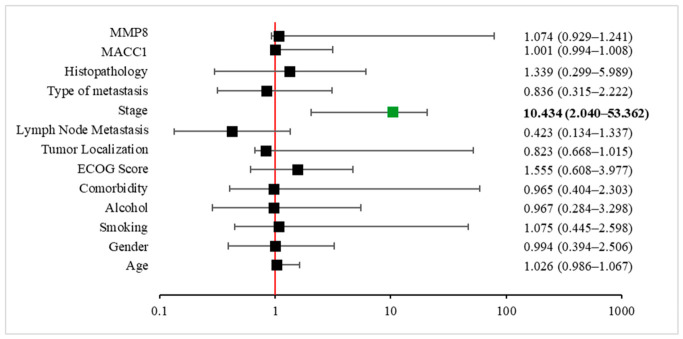
Multivariate Cox proportional hazards analysis of factors associated with overall survival. Forest plot illustrating HRs with 95% CIs for clinicopathological and molecular variables included in the multivariate Cox regression model. The vertical reference line represents HR = 1, and horizontal bars indicate 95% CIs. The green box indicates a statistically significant parameter.

Multivariate Cox proportional hazards regression analysis showed that tumor stage was the only independent predictor of overall survival (Figure 4). Patients with advanced stage disease had a significantly increased risk of mortality (HR = 10.43, 95% CI: 2.04–53.36, *p* = 0.005), as illustrated in the forest plot, where the confidence interval remained clearly above unity. In contrast, other clinicopathological variables—including age, gender, smoking status, alcohol consumption, comorbidity, ECOG performance status, tumor localization, lymph node metastasis, type of metastasis, histopathological subtype, and molecular markers (MACC1 and MMP8) were not significantly associated with OS in the multivariate model (all *p* > 0.05). The forest plot (Figure 4) highlights that disease stage is the dominant and independent prognostic factor affecting survival in this cohort, whereas other clinical and molecular parameters do not retain significance after adjustment for confounding variables.

In the multivariate linear regression analysis, tumor localization and disease stage emerged as significant independent predictors of the outcome variable (Table 3). Tumor localization was positively associated with the outcome (β = 0.250, *p* = 0.001), whereas stage demonstrated a significant inverse relationship (β = −0.420, *p* = 0.002), indicating that increasing disease stage was associated with a decrease in the dependent variable. Lymph node metastasis showed a trend toward significance (β = 0.154, *p* = 0.070), although it did not reach statistical significance. Other clinicopathological variables, including age, gender, smoking status, alcohol consumption, comorbidity, ECOG performance status, type of metastasis, and histopathological subtype, were not significantly associated with the outcome (all *p* > 0.05). Similarly, molecular markers MALAT1 and MMP8 did not demonstrate independent predictive value in the multivariate model (*p* = 0.916 and *p* = 0.238, respectively). Collinearity diagnostics revealed moderate multicollinearity among certain variables, particularly MALAT1 and MMP8 (VIF ≈ 5), which may have influenced the stability of the regression estimates.

To further explore the independent prognostic roles of molecular markers, separate multivariate Cox regression models were constructed including either MMP8 or MACC1 alongside clinicopathological variables. In the model incorporating MMP8, three variables emerged as significant predictors of overall survival. Tumor stage remained a strong independent prognostic factor (HR = 10.19, 95% CI: 2.00–51.87, *p* = 0.005). Notably, MMP8 expression was also independently associated with survival (HR = 1.09, 95% CI: 1.00–1.19, *p* = 0.046), indicating that increased MMP8 levels were linked to a higher risk of mortality. In addition, tumor localization reached borderline statistical significance (HR = 0.82, 95% CI: 0.67–1.00, *p* = 0.050), suggesting a potential site-dependent effect on survival. Other variables included in the model were not statistically significant (all *p* > 0.05). In contrast, in the model including MACC1, tumor stage again remained the only robust independent prognostic factor (HR = 12.14, 95% CI: 2.42–61.00, *p* = 0.002). Although MACC1 expression showed a trend toward significance (HR = 1.004, 95% CI: 0.999–1.008, *p* = 0.093), it did not reach statistical significance. Similarly, tumor localization demonstrated a near-significant association (HR = 0.83, 95% CI: 0.68–1.02, *p* = 0.078), while all other clinicopathological variables remained non-significant.

## 4. Discussion

In the present study, we investigated the prognostic significance of MACC1 and MMP8 expression in CRC patients. Specifically, MACC1 overexpression has been correlated with increased tumor growth, invasion, and migration in numerous cancer types, including CRC [20,21,22,23]. Conversely, MMP8, a proteolytic enzyme involved in extracellular matrix degradation, has shown conflicting prognostic value in CRC, with some studies indicating that high serum levels of MMP8 correlate with decreased survival and systemic inflammation, particularly in colon cancer and left-sided tumors [24,25]. However, other investigations have not observed a consistent correlation between MMP8 serum levels and tumor malignancy, highlighting the necessity for further research into its precise role in CRC carcinogenesis [26]. This ambiguity surrounding MMP8’s precise role underscores the need for comprehensive investigations into its gene expression profiles and their correlation with various clinicopathological parameters in CRC [27,28]. Therefore, this study aims to assess the individual and combined prognostic significance of MACC1 and MMP8 serum levels, alongside established clinicopathological factors, to develop a more robust predictive model for CRC patient outcomes. This includes an examination of how these molecular markers interact with established factors such as tumor stage, histological grade, and lymph node involvement to refine prognostic predictions and potentially guide personalized treatment strategies. Furthermore, a deeper understanding of the molecular interplay between MACC1, MMP8, and their regulatory pathways could reveal novel therapeutic targets for precision medicine in CRC. Another lncRNA, Metastasis-associated lung adenocarcinoma transcript 1, initially identified as a predictive biomarker of metastasis in non-small cell lung cancer, has subsequently been found to hold prognostic significance in CRC patients [29]. The AUC value obtained in the present study (~0.72) indicates a moderate discriminatory ability of the biomarker to distinguish CRC patients from healthy individuals. Although this level of performance is not sufficient for use as a standalone diagnostic tool, it is comparable to other screening methods reported in the literature, such as fecal immunochemical tests and certain single-protein biomarkers [30,31]. These findings suggest that the marker may have potential utility not as an independent diagnostic test, but as part of multi-marker panels designed to improve overall diagnostic accuracy.

A strong positive correlation between MACC1 and MMP8 serum levels was observed in this study, suggesting that these two molecules may participate in related biological processes. This association may be relevant to cancer pathogenesis, particularly given the regulatory influence of MACC1 on MMPs, as previously reported [32,33,34,35,36,37]. The role of MMP in cancer progression has been widely studied, although their prognostic significance differs among family members and tumor types. Pan-cancer analyses have shown that several MMPs, including MMP1, MMP9, MMP10, MMP11, and MMP13, are frequently upregulated and may have diagnostic or prognostic relevance [38]. In this context, the observed positive correlation between MACC1 and MMP8 in the current cohort may reflect a shared regulatory pattern; however, this relationship should be interpreted with caution. The role of MMP8 in cancer remains less clearly defined than that of other MMP family members, with evidence suggesting context-dependent effects. In our study, elevated serum MMP8 levels were found to be associated with tumor progression and metastasis risk, consistent with the existing literature [24,25]. However, caution is warranted when interpreting the biological source of MMP8, as this protease is primarily expressed and released by neutrophils [24,39]. Serum MMP8 levels have been shown to positively correlate with increased peripheral neutrophil counts and may rise as part of the acute-phase inflammatory response [25,39]. Therefore, the observed elevation in serum MMP8 may reflect not only tumor cell activity but also the host’s systemic inflammatory response to the tumor or neutrophil infiltration secondary to tumor necrosis [26,39]. Similarly, although serum MACC1 levels are considered a strong predictor of metastasis and poor prognosis, this marker has also been reported to be elevated in other cancer types and various pathological conditions [40,41]. This suggests that both markers should be interpreted not as tumor-specific proteins, but rather as indicators reflecting tumor–microenvironment interactions and systemic biological alterations [15,22]. While some studies, particularly in breast cancer, have associated MMP8 protein levels with a more favorable prognosis, others have reported increased expression in gastrointestinal and lung cancers, raising the possibility of divergent functions depending on tumor type [42,43]. These findings highlight the complexity of MMP8-related mechanisms and indicate that further studies are needed to clarify its interaction with MACC1 and its potential role within the tumor microenvironment.

These findings suggesting a context-dependent role for MACC1 in tumor progression, with higher serum levels potentially exerting a suppressive effect on metastasis in certain contexts. However, its association with earlier tumor stages highlights the need for further investigation into its mechanistic involvement in tumor initiation or early progression. In contrast, the limited association of MMP8 serum level mainly with tumor localization suggests a potentially tissue-specific regulatory or effector function for this protease in colorectal carcinogenesis. Further evaluation of additional clinical parameters, such as tumor differentiation or vascular invasion, could clarify the specific contextual roles of both MACC1 and MMP8 [25]. Additional research into the molecular pathways underlying these differential associations, particularly regarding MACC1’s paradoxical role in metastasis and tumor stage, is necessary to fully determine their clinical significance. Given the observed associations, especially the inverse relationship between MACC1 serum level and metastatic status, future studies should focus on the regulatory mechanisms controlling MACC1 serum level and its functional impact on cellular processes critical to metastasis, such as cell migration, invasion, and epithelial–mesenchymal transition. This is particularly relevant because MACC1 has been reported to be upregulated in CRC tissues and associated with advanced TNM stages, recurrence, and poor overall survival in some studies [23,44,45,46,47]. In CRC, specifically, MACC1 knockdown dramatically inhibits cellular proliferation, migration, invasion, colony formation, and tumorigenesis in vitro and in vivo, while simultaneously inducing apoptosis in CRC cells [48]. This multifaceted influence underscores MACC1’s complex involvement in CRC pathophysiology, often involving interactions with β-catenin and its downstream targets like c-Myc and cyclin D1 [49]. These discrepancies highlight the need for a comprehensive understanding of MACC1’s diverse regulatory mechanisms and its downstream effectors in different CRC subtypes and clinical settings.

In this cohort, survival outcomes were primarily determined by established clinical factors, particularly tumor stage, metastatic status, and performance status. A high proportion of patients were diagnosed at advanced stages, likely contributing to poorer overall survival and possibly reflecting delays in diagnosis or limited screening. Consistent with previous evidence, the presence of distant metastasis was one of the strongest predictors of survival, while patients without metastasis had significantly better outcomes. Similarly, poorer ECOG performance status was associated with reduced survival, underscoring its clinical relevance. Tumor localization and surgical approach were associated with survival; however, these findings may reflect underlying disease characteristics and treatment selection rather than independent prognostic effects. In contrast, demographic variables and histopathological features were not significantly associated with survival in this cohort. Notably, MACC1 and MMP8 serum levels did not show a significant prognostic impact despite their biological relevance, which may be due to sample size, cohort heterogeneity, or their context-dependent roles in CRC biology. Overall, these findings emphasize the dominant role of established clinicopathological factors in survival prediction and highlight the need for larger, well-characterized cohorts to clarify the clinical utility of emerging molecular biomarkers in CRC.

This study evaluated clinicopathological and molecular determinants of survival, identifying tumor stage as the strongest independent predictor of overall survival. Advanced-stage disease was consistently associated with increased mortality across all models, underscoring its central role in prognostic stratification. When molecular variables were assessed separately, MMP8 serum level emerged as an additional independent prognostic factor, with higher levels associated with increased mortality risk. This finding is consistent with previous reports linking elevated MMP8 levels to adverse outcomes [24,50]. However, conflicting evidence suggests a context-dependent role for MMP8, with some studies reporting protective effects in certain tumor types [51], indicating that its biological impact may vary depending on the tumor microenvironment. In contrast, MACC1 serum level did not retain independent prognostic significance in multivariate analysis, despite showing a trend toward association with survival. This suggests that, although MACC1 may contribute to CRC biology, its prognostic value appears limited when adjusted for established clinical parameters.

Several limitations of this study should be acknowledged. First, due to the retrospective design, causal relationships could not be definitively established, so the findings should be interpreted as associative. The study was conducted at a single center, and the study population represents a specific geographic and demographic group; thus, the generalizability of the results may be limited. Although the sample size was determined by power analysis, the statistical power may have been insufficient for certain subgroup analyses. The use of serum-based measurements precluded evaluation of tissue-level expression and interactions within the tumor microenvironment, limiting mechanistic interpretation. A significant limitation of the present study is that the control group consisted solely of healthy individuals. In clinical practice, colorectal cancer must be differentiated not only from healthy subjects but also from inflammatory and benign conditions presenting with similar clinical features, such as Crohn’s disease, ulcerative colitis, and benign polyps [52,53]. Therefore, to better determine the true clinical specificity of these biomarkers, future studies should include such patient populations. In addition, biomarkers were assessed at a single time point, preventing evaluation of their dynamic changes and potential role in treatment response over time. The limited prognostic impact of the molecular markers in multivariate analyses suggests that their clinical utility requires further validation in larger, prospective, multicenter studies. Finally, not all potential confounding factors may have been fully controlled, and missing data for certain clinical variables might have influenced the results.

## 5. Conclusions

In conclusion, this study offers a comprehensive evaluation of the prognostic significance of MACC1 and MMP8 serum levels in CRC cohort. A significant positive correlation between MACC1 and MMP8 levels was found, indicating a potential shared regulatory or biological interaction. Although both biomarkers showed moderate diagnostic performance, their overall impact on survival was limited after adjustment for established clinicopathological factors. These results indicate that, while MACC1 and MMP8 may have biological and potential diagnostic relevance in CRC, their prognostic value is secondary to established clinical parameters. Further large-scale, prospective, and mechanistic studies are necessary to clarify their roles and determine their potential integration into clinical practice.

## Figures and Tables

**Table 1:** Chi-square analysis of MACC1 and MMP8 expression levels with clinicopathological parameters.

Clinical Parameters		MACC1		*p* Value	MMP8		*p* Value
		Low	High		Low	High	
Age	<50	15	7	0.063	14	8	0.143
	>50	55	63		55	63	
Gender	Male	46	50	0.466	48	48	0.803
	Female	24	20		21	23	
Smoking	Yes	31	30	0.865	31	30	0.75
	No	39	40		38	41	
Alcohol	Yes	16	10	0.192	13	13	0.936
	No	54	60		56	58	
Comorbidity	Yes	25	31	0.301	24	32	0.214
	No	45	39		45	39	
Family History	Yes	25	18	0.2	24	19	0.304
	No	45	52		45	52	
CRC history of family	Yes	8	6	0.573	7	7	0.955
	No	62	64		62	64	
ECOG Score	0	35	36	0.515	31	40	0.335
	1	29	32		34	27	
	2	5	2		3	4	
	3	1	0		1	0	
Tumor Localization cat.	Colon	39	42	0.608	32	49	***0.007***
	Rectum	31	28		37	22	
Operation	LAR	27	33	0.232	31	29	0.39
	Miles	10	3		9	4	
	Colectomy	28	28		25	31	
	Palliative surgery	5	5		4	6	
	Total proctocolectomy	0	1		0	1	
Obstruction	Yes	7	10	0.438	6	11	0.218
	No	63	60		63	60	
Lymph Node Metastasis	No	54	46	0.134	51	49	0.521
	Yes	16	24		18	22	
Metastasis	Yes	36	23	0.026	30	29	0.752
	No	34	47		39	42	
Localization of metastasis	No	34	47	0.039	39	42	0.421
	Liver	22	18		18	22	
	Other	14	5		12	7	
Type of metastasis	No	34	47	0.062	39	42	0.207
	Synchronous	19	15		14	20	
	Metachronous	17	8		16	9	
Stage	II	5	12	0.044	6	11	0.482
	III	29	35		33	31	
	IV	36	23		30	29	
Early/Late Stage	Early	4	8	0.366	4	8	0.367
	Late	66	62		65	63	
Histopathology	Adeno	66	63	0.346	63	66	0.716
	Mucinose	4	7		6	5	

**Table 2:** Kaplan–Meier analysis of clinicopathological parameters associated with overall survival.

Clinical Parameters		Estimate ^a^	Std. Error	95% CI		*p* Value
				Lower	Upper	
Age	<50	22.126	1.424	19.335	24.917	0.474
	>50	26.846	1.198	24.499	29.194	
Gender	Male	26.294	1.342	23.664	28.924	0.758
	Female	26.697	1.902	22.969	30.426	
Smoking	Yes	25.731	1.707	22.385	29.078	0.86
	No	27.312	1.428	24.514	30.111	
Alcohol	Yes	28.525	2.066	24.476	32.574	0.321
	No	26.308	1.297	23.766	28.851	
Comorbidity	Yes	20.685	1.122	18.486	22.885	0.842
	No	26.588	1.487	23.673	29.503	
Family History	Yes	22.173	0.992	20.228	24.118	0.208
	No	25.927	1.434	23.117	28.737	
CRC history of family	Yes	19.108	1.293	16.574	21.643	0.526
	No	26.676	1.193	24.337	29.015	
ECOG Score	0	23.179	0.879	21.457	24.902	0.001
	1	25.729	1.692	22.412	29.046	
	2	9.571	2.776	4.130	15.013	
	3	13.000	0.000	13.000	13.000	
Tumor Localization cat.	Colon	24.678	1.634	21.476	27.881	0.029
	Rectum	29.148	1.266	26.667	31.630	
Obstruction	Yes	20.667	2.013	16.722	24.612	0.595
	No	27.113	1.220	24.722	29.504	
Lymph Node Metastasis	No	24.827	1.385	22.111	27.542	0.05
	Yes	30.482	1.445	27.650	33.314	
Metastasis	Yes	15.859	1.383	13.148	18.571	<0.001
	No	32.484	0.738	31.039	33.930	
Localization of metastasis	No	32.484	0.738	31.039	33.930	<0.001
	Liver	15.352	1.680	12.059	18.645	
	Other	16.433	2.306	11.914	20.952	
Type of metastasis	No	32.484	0.738	31.039	33.930	<0.001
	Synchronous	15.402	1.790	11.893	18.912	
	Metachronous	14.981	1.730	11.589	18.372	
Histopathology	Adeno	27.017	1.172	24.720	29.314	0.643
	Mucinose	18.465	2.707	13.158	23.771	
MACC1 status	Low	27.063	1.482	24.158	29.969	0.341
	High	26.178	1.573	23.095	29.261	
MMP8 status	Low	22.122	0.976	20.210	24.035	0.295
	High	26.066	1.599	22.933	29.200	

^a^ Estimation is limited to the largest survival time if it is censored.

**Table 3:** Multivariate Linear Regression Analysis of Factors Associated with tumor localization and stage.

Model	Unstandardized Coefficients		Standardized Coefficients	t	Sig.	Collinearity Statistics	
	B	Std. Error	Beta			Tolerance	VIF
(Constant)	2.654	0.432		6.143	0.000		
Age	−0.001	0.003	−0.018	−0.218	0.828	0.728	1.373
Gender	−0.029	0.075	−0.033	−0.390	0.697	0.736	1.360
Smoking	0.032	0.073	0.039	0.444	0.658	0.669	1.496
Alcohol	−0.020	0.088	−0.018	−0.223	0.824	0.748	1.338
Comorbidity	0.041	0.072	0.048	0.566	0.573	0.711	1.407
ECOG Score	−0.087	0.065	−0.105	−1.331	0.186	0.826	1.210
Tumor Localization	0.050	0.015	0.250	3.310	0.001	0.897	1.115
Lymph Node Metastasis	0.142	0.078	0.154	1.827	0.070	0.717	1.395
Type of metastasis	−0.002	0.076	−0.004	−0.025	0.980	0.255	3.917
Stage	−0.259	0.082	−0.420	−3.148	0.002	0.288	3.477
Histopathology	−0.110	0.116	−0.071	−0.947	0.345	0.904	1.107
MALAT1	−0.815	0.001	−0.017	−0.105	0.916	0.194	4.957
MMP8	−0.020	0.017	−0.192	−1.185	0.238	0.195	4.921

## Data Availability

The original contributions presented in this study are included in the article. Further inquiries can be directed to the corresponding author.
